# Gait Speed in Older Adults and Cognitive Health

**DOI:** 10.1093/geroni/igaf122.114

**Published:** 2025-12-31

## Abstract

Mounting evidence supports the relationship between Gait speed and cognition. This session will add to the science on this topic of the link of gait speed to cognition.

